# ICSI versus Conventional IVF in Women Aged 40 Years or More and Unexplained Infertility: A Retrospective Evaluation of 685 Cycles with Propensity Score Model

**DOI:** 10.3390/jcm8101694

**Published:** 2019-10-16

**Authors:** Gianluca Gennarelli, Andrea Carosso, Stefano Canosa, Claudia Filippini, Sara Cesarano, Carlotta Scarafia, Nicole Brunod, Alberto Revelli, Chiara Benedetto

**Affiliations:** 1 Obstetrics and Gynecology 1U, Physiopathology of Reproduction and IVF Unit, Department of Surgical Sciences, Sant’Anna Hospital, University of Torino, 10042 Torino, Italy; andrea88.carosso@gmail.com (A.C.); s.canosa88@gmail.com (S.C.); sara.cesarano2@gmail.com (S.C.); carlotta.scarafia@gmail.com (C.S.); nicole.brunod@gmail.com (N.B.); alberto.revelli@unito.it (A.R.); chiara.benedetto@unito.it (C.B.); 2 Department of Surgical Sciences, University of Torino, 10042 Torino, Italy; claudia.filippini@unito.it

**Keywords:** in vitro fertilization, ICSI, IVF, non-male factor infertility, cumulative live birth rates

## Abstract

This study compared the cumulative live birth rates following Intracytoplasmic sperm injection (ICSI) versus conventional in vitro fertilization (cIVF) in women aged 40 years or more and unexplained infertility. A cohort of 685 women undergoing either autologous conventional IVF or ICSI was retrospectively analyzed. The effects of conventional IVF or ICSI procedure on cumulative pregnancy and live birth rates were evaluated in univariate and in multivariable analysis. In order to reduce potential differences between women undergoing either IVF or ICSI and to obtain unbiased estimation of the treatment effect, propensity score was estimated. ICSI was performed in 307 couples (ICSI group), whereas cIVF was performed in 297 couples (cIVF group), resulting in 45 and 43 live deliveries, respectively. No differences were observed in morphological embryo quality, in the number of cleavage stage embryos, in the number of transferred embryos, and in the number of vitrified embryos. As for the clinical outcome, no differences were observed in pregnancy rate, cumulative pregnancy rate, live birth rate, cumulative live birth rate, and abortion rate. The present results suggest that ICSI is not associated with increased likelihood of a live birth for unexplained, non-male factor infertility, in women aged 40 years or more.

## 1. Introduction

Intracytoplasmic sperm injection (ICSI) was introduced in 1992 in order to treat infertility in couples with severe male factor [1]. Since then, the use of ICSI as an alternative to conventional in vitro fertilization (cIVF) has increased steadily, with a proportion rising worldwide from 47.6% of all IVF cycles in 2000 to 66% in 2010, and exceeding 90% in some countries [2,3]. In Italy, the use of ICSI was 6 times more frequent than that of cIVF in 2014 [4].

Interestingly enough, the largest increase in ICSI application has been observed among couples without male factor infertility (from 15% in 1996 to 67% in 2012) [5]. Indeed, ICSI has been suggested as the elective treatment for couples with unexplained infertility [6]. Furthermore, ICSI has been used more and more as a general routine for IVF, due to the belief that it could prevent up to 30% of fertilization failures [7]. However, so far there is no clear evidence that ICSI leads to better reproductive outcomes in non-male infertility, compared to cIVF. A previous Cochrane review of cIVF versus ICSI in couples with infertility not related to a male factor did not report differences in pregnancy rates (PR). However, the review did not include in the analysis randomized data with live birth rates (LBR) [8]. More recent data seem to confirm that cIVF and ICSI result in similar reproductive outcomes in both non-male infertility [9] and patients with a poor response to ovarian stimulation and few oocytes retrieved [10,11].

In spite of these results, ICSI has been suggested in alternative to cIVF for women with age-related infertility, whose proportion is dramatically increased over the last decades [12]. As a matter of fact, oocytes retrieved from older women are often of lower quality [13], and cIVF could hypothetically result in decreased fertilization rates in these patients, due to problems in sperm-oocyte interaction [14]. Therefore, for many clinicians, ICSI should be the preferred method in this population, but such a choice is not based on solid grounds, as clinical evidence is currently lacking.

The purpose of the present study was to compare ICSI and cIVF in women aged 40 years or more, affected by unexplained infertility. The primary end-point was the cumulative live birth rate, including transfers of both fresh and frozen embryos. Secondary end points were fertilization rate, embryo morphological score, implantation rate, abortion rate.

## 2. Materials and Methods

### 2.1. Patient Population 

In this retrospective analysis we included a cohort of women aged ≥ 40 years, with unexplained infertility, undergoing either cIVF or ICSI in the years 2012–2018, at a single academic IVF unit. Only autologous cycles were considered. All women included in the study had ovulatory cycles and at least patency of one fallopian tube at sonosalpingography (SSG), whereas all male partners had normal basic semen parameters according to the indications of WHO 2010. Exclusion criteria were: Any known cause of female infertility (i.e. previous history of pelvic inflammatory disease, positive anti-Chlamydia IgG, endometriosis, anovulation, etc), female body mass index (BMI: Kg/m^2^) > 32 kg/m^2^, early follicular phase follicle stimulating hormone (FSH) > 20 UI/l and/or anti-Mullerian hormone (AMH) < 0.1, cycles with pre-implantation genetic diagnosis, and combined insemination (cIVF/ICSI) of sibling oocytes. The study was performed in accordance with the Helsinki Declaration and with approval of the Institutional Review Board. Since this was a retrospective analysis of data routinely collected from treatments and patients, a specific informed consensus was not required, according to the rules of the Department of Ob&Gyn of S. Anna hospital.

### 2.2. ART Procedure

Controlled ovarian stimulation (COS) was performed either with recombinant FSH (rFSH), human Menopausal Gonadotropin (HP-hMG) or rFSH plus recombinant luteinizing hormone (rLH), under pituitary suppression. The choice of the starting gonadotropin dose was based on age, BMI, antral follicular count (AFC), AMH concentrations, as well as on the response to previous COS. In the absence of any pre-fixed criteria, the COS regimen (type of protocol and type of medication) was decided and prescribed by different physicians of the IVF unit, according to their own clinical experience, as per real-life clinical practice.

Both long and short protocols were used. The long protocol was performed administering buserelin (Suprefact®, Hoechst, Frankfurt, Germany; 900 mcg/d intranasally) starting from the late luteal phase of the previous cycle. After approximately two weeks, pituitary suppression was verified (appearance of a menstrual bleeding, serum estradiol < 50 pg/mL, endometrial thickness <3 mm) before starting COS. 

In the short protocol, either the GnRH-antagonist cetrorelix (Cetrotide®, Merck, Darmstadt, Germany) or ganirelix (Orgalutran ®, Merck Sharp & Dome, Kenilworth, NJ, USA) were started at a subcutaneous dose of 0.25 mg/d according to a flexible schedule, when at least one follicle ≥ 12 mm in mean diameter was observed at ultrasound (US).

COS was monitored by serial transvaginal US and serum estradiol (E2) measurements performed every second day from stimulation day 6–7. COS continued until at least two follicles reached 18 mm in mean diameter, when ovulation was triggered by injecting either 10,000 international units (IU) of hCG (Gonasi HP®, IBSA, Lugano, Switzerland) or 250 mcg of rHCG (Ovitrelle®, Merck, Darmstadt, Germany) subcutaneously. US-guided transvaginal oocyte aspiration (OPU) was performed approximately 36–37 h after hCG administration, under local anesthesia (paracervical block). Oocytes were immediately recovered from the follicular fluid, then washed in buffered medium and stored until fertilization procedure.

### 2.3. Preparation of Semen Samples and in Vitro Fertilization

Semen samples were examined to assess sperm concentration, motility, and morphology according to the World Health Organization guidelines [15]. Samples were then prepared by density gradient centrifugation in order to select motile, morphologically normal spermatozoa.

The embryologist on duty the day of OPU decided the insemination method in agreement with the physician in charge of the treatment. According to all physicians working in the unit, the decision was oriented mainly by two parameters: previous attempts and duration of infertility. However, criteria varied among doctors, whereas they were not applied in all cases uniformly. Given the lack of shared clinical guidelines, such an approach to treatment in this particular group of patients is reported as rather common [16].

Conventional IVF or ICSI were performed on all available oocytes within 4 hours after oocytes collection and, after 16–18 hours of incubation in controlled atmosphere, the occurrence of normal fertilization was assessed. 

### 2.4. Embryo Selection and Transfer

In total, 1787 embryos were obtained in 685 IVF cycles. Zygotes were placed in pools in 4-wells dish (Thermo Scientific, Roskilde, Denmark) and embryos were cultured in pre-equilibrated Cleavage medium (Cook) overlain with mineral oil using incubators (Minc, COOK, Bloomington, IN, USA) in a gas phase consisting of 5% O_2_, 6–7% CO_2_, balanced with N_2_.

All cleaved embryos were morphologically evaluated under a conventional stereomicroscope, using the IMCS score by Holte [17]. A total number of 900 embryos were selected on day 2 or 3 for transfer in uterus (1.3 ± 0.8 per patient), which was performed using a soft catheter, under ultrasound guidance. According to the policy of our IVF unit during the time period under study, one or two embryos were transferred on day 2/3 post-fertilization. The remaining embryos (if any) were kept in culture until the blastocyst stage. Only embryos reaching the blastocyst stage could be vitrified. The luteal phase was supported by administering 180 mg/day natural progesterone (Crinone 8®, Merck, Darmstadt, Germany) for 15 days. 

Pregnancy was assessed by serum hCG assay after 15 days from embryo transfer (ET) and then confirmed if at least one gestational sac was visualized on transvaginal US after two further weeks. Only cases with US confirmation of pregnancy were counted in the calculation of pregnancy rate, whereas biochemical pregnancies were not considered.

### 2.5. Statistical Analysis

Continuous data are presented as mean and standard deviation (SD), while categorical data are presented as rate and proportion. 

The effects of cIVF or ICSI procedure on pregnancy and live birth rates were evaluated in univariate and in multivariable analysis. In univariate analysis, comparisons of continuous data were performed using either the unpaired Student’s t-test or Wilcoxon Mann-Whitney test, according to data distribution; comparisons of categorical data were performed using either Chi-square test or Fisher’s exact test, as appropriate. In multivariable analysis outcomes were evaluated using two different approach: conventional multivariable analysis and analysis using Propensity Score.

### 2.6. Propensity Score

In 1983, Rosenbaum and Rubin introduced the Propensity Score (PS) as a means to control for confounding in observational studies [18]. The PS combines the information from all measured confounders into one score and can be seen as a one-dimensional variable that summarizes the multidimensional pretreatment covariates. Conditional on the PS, the treatment and the control groups are expected to have the same distribution of measured confounders and therefore allows for a direct comparison between groups [19].

In order to reduce potential differences between women undergoing either cIVF or ICSI and to obtain unbiased estimation of the treatment effect, PS was estimated. In this study PS is the probability of receiving IVF procedure conditionally on a priori selected variables. A multivariable logistic regression model was performed with presence of cIVF or ICSI procedure as the dependent variable. Variables included in the propensity score model were years of infertility, previous ART treatments, female age, smoke, AFC, AMH, FSH, BMI, endometrial thickness, partner’s age, sperm concentration, sperm progressive motility, proportion of spermatozoa with normal morphology, embryo morphological score and number of embryos transferred.

All outcomes (pregnancy rate, cumulative pregnancy rate, live birth rate, cumulative live birth rate) have been evaluated with conventional covariate adjustment and with PS adjustment; in both approach results were reported as odds ratios (OR) with a 95% confidence intervals (95% CI).

All statistical tests were two-sided and *p* values of 0.05 or less were considered statistically significant. All analysis was conducted using the SAS version 9.4 (SAS Institute, Cary, NC, USA) software package.

## 3. Results

Among the 685 couples who met the inclusion criteria, 81 women did not obtain any mature oocyte at OPU and were excluded from further analysis. The number of couples who failed to recover any oocyte at ovum pick-up did not differ between cIVF and ICSI groups. 

At least one mature oocyte was retrieved in the remaining 604 couples. Baseline characteristics of the women included in the analysis are presented in Table 1. 

ICSI was performed for 307 couples (ICSI group), whereas cIVF was performed for 297 couples (cIVF group). All variables were similar between the cIVF and ICSI groups except for the years of infertility and a history of previous ART treatments, which were both higher in the ICSI group. In total, 388 patients (*n* = 217 in cIVF group, *n* = 169 in ICSI group) had never undergone an IVF treatment before. Also, in this sub-group of patients, basal characteristics were similar except for the years of infertility (see Table S1). Variables related to cycle outcome are shown in Table 2. 

No differences were registered in the dose of exogenous FSH and LH, or in the thickness of the endometrium at the end of COS. 

The number of retrieved oocytes and the absolute number of mature (MII) oocytes did not differ between the groups; however, the proportion of MII oocytes was higher in the IVF group, so was the overall fertilization rate. When only MII oocytes were considered, the fertilization rate did not differ between the study groups. 

No differences were observed in morphological embryo quality, in the number of cleavage stage embryos, in the number of transferred embryos, and in the number of vitrified embryos. As for the clinical outcome, no differences were observed in pregnancy rate, cumulative pregnancy rate, live birth rate, cumulative live birth rate, and abortion rate. As shown in Table 3, the results did not change even after adjusting for several confounding variables and applying the Propensity Score.

In order to account for the potential confounding factor deriving from the number of previous treatments, a sub-analysis including only patients at their first treatment was carried out. The results are comparable to those from the general population examined and, even after correction with the propensity score, it was not possible to demonstrate the superiority of one technique over the other in terms of cumulative pregnancy rates and cumulative live birth rates (see Table 4 and Table 5).

## 4. Discussion

Notwithstanding the lack of evidence-based guidelines, the routine use of ICSI has been proposed for women in advanced reproductive age regardless the cause of infertility, with the rationale that ICSI would be associated with a reduced likelihood of fertilization failure [20,21]. A higher fertilization rate would result in an increased number of available embryos, a crucial issue especially in patients with a low ovarian reserve and low oocyte yield, which is often the case in older women. 

The issue is not of minor relevance. Indeed the number of women of advanced reproductive age is constantly increasing worldwide: according to the last ESHRE report, in 2014 approximately 17% of cIVF cycles and 20% of ICSI cycles were performed in women aged 40 years or older [4], whereas more than 35% of women attending fertility clinics in Italy in 2016 were 40 years or older [22]. Data from US report a similar picture, as in 2015, 16% of ART cycles were carried out among women aged 41–44 years [23].

Oocytes of older women were theorized to have structural defects that might reduce the fertilization potential; one of the putative mechanisms suggested for a decreased fertilization rate in older women would be the zona pellucida hardening. However, limited data from women at the extreme of reproductive age range (45 years or more) with a normal partner did not confirm this hypothesis [24].

Recently, the results of a systematic review and meta-analysis of couples with well-defined unexplained infertility suggested the use of ICSI instead of cIVF, regardless of female age, in order to increase the fertilization rate per retrieved oocyte [6]. That meta-analysis included 11 studies, which reported data on sibling oocytes randomly split to be inseminated either with ICSI or cIVF. However, only four of the included studies reported pregnancy outcomes by insemination method. Furthermore, as the authors pointed out, the studies used different criteria for selecting embryos for transfer, and the patient groups were small and not comparable among different studies. Even more important, the studies selected for the meta-analysis included patients with an average number of retrieved oocytes between 10.8 and 16.3: this would suggest that the included women represented a subgroup of patients with good prognosis. 

At variance with those results, more recent data on patients of various ages reported comparable or even higher fertilization rates per retrieved oocyte for cIVF than ICSI [9,10,16,25]. The largest population-based cohort study published so far, which included women distributed on a wide age range belonging to couples with non-male factor infertility, resulted in a similar cumulative live birth rate (up to 6 treatment cycles) between cIVF and ICSI [9]. However, the results were not stratified according to the woman age range. Another recent study in women aged between 40–43 years treated for non-male factor infertility found that cIVF resulted in a higher fertilization rate per retrieved oocyte compared with ICSI (57% versus 52%). This result, however, was partially explained by the larger number of mature oocytes (MII) in the IVF group [16].

In this panorama, the present study is the first one investigating the *cumulative* live birth rate in women aged 40 years or more with unexplained infertility, following either cIVF or ICSI. Our results show that in this specific group of patients, cIVF and ICSI result in similar cumulative live birth rates once all viable embryos are transferred. The insemination technique in our study, in fact, did not affect embryo quality according to the morphological score [17], nor it affected the cumulative pregnancy rate, the cumulative live birth rate and the abortion rate. Whereas the results related to these clinical variables should be interpreted with caution, due to the limited number of observations, they are in line with recent data from other groups [9,16], corroborating the notion that this particular subgroup of patients would not benefit from ICSI, in the absence of a male-factor.

In our study, a higher proportion (but not the absolute number) of MII oocytes, together with a higher fertilization rate per retrieved oocyte, were observed in cIVF group. This finding cannot be explained by differences in pre-treatment clinical parameters, nor in the COS protocols, since all the considered variables were comparable between the groups. An alternative explanation could be that immature oocytes elected for cIVF could have completed nuclear maturation in vitro, since in cIVF the cumulus cells are left intact, whereas in ICSI, when oocyte denudation procedure was applied, immature oocytes were immediately discharged. 

When only mature oocytes were considered, fertilization rates tended to be higher in the IVF group (*p* 0.07): this trend could not be explained by sperm quality, as the conventional semen parameters were similar in the two groups. A potential explanation could be the oocyte damage caused by the invasive procedure of microinjection [16,26,27], a mechanical injury somehow facilitated by advanced maternal age. Should this hypothesis be confirmed in larger studies, ICSI could even turn out to be contraindicated for older women in the absence of a male factor. 

In the cited study by Tannus and coworkers [16], more zygotes and more embryos to freeze were obtained in the cIVF group. Again, this probably depended on the larger number of MII oocytes in the cIVF group. Unfortunately, it is not possible to know whether the higher number of frozen embryos in the cIVF group would have resulted in higher cumulative pregnancy and live birth rates, since the study did not included thawing cycles [16]. 

A very recent retrospective cohort study from Israel in couples with non-male factor infertility [28] included women aged 35–45 years. By design, sibling oocytes were randomly assigned to either cIVF or ICSI. The fertilization rate and the top-quality embryo rate were significantly higher for the oocytes in the ICSI group (implantation rate and clinical pregnancy rate according to the method of insemination were not end-points). However, when analyzing the subgroup of patients aged 40–45 years (a similar age range to the patients enrolled in our study), differences were no longer significant, suggesting that the putative advantage of ICSI over cIVF would be limited to younger patients. 

However, even this hypothesis would be questionable according to the results of a similar recent retrospective study on a large group of women with mean age 37, and normo-spermic partners [29]. In that study, which had a design similar to ours, the fertilization rate, the number of frozen embryos, the cumulative pregnancy rate and the live birth rate were significantly lower for the couples treated with ICSI. In order to explain their findings, the authors suggested on one side a potential role of natural selection of the sperm in cIVF, on the other side a detrimental role of ICSI as a consequence of mechanical damage to both the oocyte and the sperm. 

A limitation of the present study is represented by its retrospective design. Furthermore, the choice between IVF and ICSI was somehow arbitrary, being based on both the number of previous ART treatments and the years of infertility. Evaluation of outcomes in previous treatments is a fundamental step to optimize the probability of success of an IVF treatment. However, couples do not always rely on the same center after one or more failures, and the information relating to previous treatments may not always be complete and exhaustive. The insemination method is therefore chosen without full awareness of the previous results, in terms of fertilization rates and embryo quality. Whereas this could represent a bias when analyzing the results in terms of pregnancies, a sub-analysis limited to only women with no previous treatments (*n* = 217 in cIVF group, *n* = 169 in ICSI group) confirmed the results obtained in the whole population (Table 4 and Table 5).

Strengths of the study are represented by the number of subjects included and the use of the PSM propensity score as a proxy variable that aggregates multiple confounding factors into a single dimension. Indeed, PSM allows to mimic randomized controlled trials [30]. Several authors suggest that it actually outperforms standard multivariable methods [31]. However, limitations in PSM were highlighted in previous papers [32] and since we believe that neither method was better in this context, we used both in our study. Furthermore, at variance with previous studies, basal semen parameters, according to the latest WHO manual, were similar between the IVF and ICSI study groups. Whereas it can be argued that basic semen analysis might have limited predictive value for pregnancy in couples trying to achieve natural conception and in couples undergoing assisted reproductive technologies (ARTs) [33], and several sperm functionality tests have been proposed [34], those have not yet been included in the WHO manual for the definition of normal semen.

Against this background, the safety of cIVF and ICSI should also be discussed. In a recent large population cohort study that included 308,974 births and 6163 births resulting from ART, the rate of birth defects in cIVF was similar to that in the general population, whereas the use of ICSI was associated with increased risk of birth defects (OR 1.57; 95% CI, 1.30–1.90) even after controlling for confounders [35]. More recently, there have been suggestions of an association between ICSI and an increase of congenital heart diseases [36], intellectual disability [37], autism [37,38] and higher rates of admission to a neonatal intensive care unit [39]. Although these trends are not confirmed in other studies [40] and meta-analyses [41,42], the safety issue of ICSI remains a matter of current debate.

## 5. Conclusions

The results of this study suggest that ICSI is not associated with increased likelihood of a live birth for unexplained, non-male factor infertility, in women aged 40 years or more. This finding is in line with the opinion of the Practice Committee of the ASRM that ‘there are no data to support the routine use of ICSI for non-male factor infertility [43].

Given the retrospective nature of the present and previous investigations, there is an urgent need for randomized prospective studies, in order to show whether ICSI has any advantage over cIVF, in particular in older women, or even whether the indiscriminate use of ICSI for non-male infertility could be detrimental.

## Figures and Tables

**Table 1:** Basal characteristics of the patients in the two study groups.

	cIVF (*n* = 297)	ICSI (*n* = 307)	*p*
Woman age (years)	41.1 ± 0.8	41 ± 0.8	0.9957
Years of infertility	2.8 ± 2.3	3.2 ± 2.4	0.0137
Couples with previous ART treatments (%)	80 (27%)	138 (45%)	< 0.0001
BMI (Kg/m^2^)	22.6 ± 3.3	23 ± 3.9	0.3832
AFC	9.5 ± 5.7	10.3 ± 6.1	0.0697
AMH (ng/mL)	1.6 ± 2.1	1.4 ± 1.3	0.5644
Women who smoke	33 (11.1%)	44 (14.3)	0.2354
Partner’s age (years)	41.9 ± 5.2	42.8 ± 5	0.0770
Sperm concentration (Mln/mL)	80.2 ± 34.1	80.4 ± 38.9	0.3121
Progressive motility (A+B; %)	40.5 ± 4.8	40.3 ± 4.4	0.4126
Normal morphology (%)	5.0 ± 0.8	4.9 ± 0.8	0.3036

BMI, body mass index; AFC, antral follicular count; AMH, anti-Mullerian hormone; cIVF, conventional in vitro fertilization; ICSI, Intracytoplasmic sperm injection.

**Table 2:** Cycle outcome in the two study groups (univariate analysis).

	cIVF (*n* = 297)	ICSI (*n* = 307)	*P*
Total FSH dose (IU)	2725 ± 1172	2816 ± 1287	0.3181
Mean FSH dose (IU)	265 ± 83	267 ± 87	0.9545
Total LH dose (IU)	1470 ± 767	1587± 892	0.5842
Mean LH dose (IU)	188 ± 89	184 ± 61	0.7537
Endometrial thickness (mm)	10.1 ± 2.4	9.9 ± 2.1	0.4445
Retrieved oocytes	5.8 ± 4.2	6.3 ± 4.6	0.2816
Mature (MII) oocytes	4.9 ± 3.4	4.9 ± 3.6	0.7006
MII/retrieved oocytes (%)	87.1	80,7	< 0.001
Fertilized oocytes	3.5 ± 2.8	3.2 ± 2.6	0.1701
Fertilization rate (%)	60,7 ± 28,3	52,1 ± 28,1	< 0.01
No fertilization (%)	6,7 (20/297)	9.4 (29/307)	0,2222
Cleaved embryos	3 ± 2.7	2.9 ± 2.5	0.8834
Transferred embryos	1.5 ± 0.7	1.4 ± 0.8	0.0899
Frozen embryos	0.4 ± 1	0.4 ± 1	0.6184
Mean embryo score	7.9 ± 1.8	7.9 ± 1.8	0.4458
PR/ET *n*.(%)	64 (10.4%)	63 (10.6%)	0.7567
Cumulative PR/OPU *n*. (%)	73 (12%)	72 (11.9%)	0.7459
LBR/ET *n*.(%)	38 (6.3%)	40 (6.6%)	0.9315
Cumulative LBR/OPU *n*.(%)	45 (7.5%)	43 (7.1%)	0.6901
Abortion rate *n*.(%)	28 (38.3%)	29 (40.3%)	0.7

FSH, follicle stimulating hormone; OPU, oocytes pick up; IU, international units; LH, luteinizing hormone; PR, pregnancy rate; ET, embryo transfer; LBR, live birth rate

**Table 3:** Cycle outcome in the two study groups (univariate analysis, multivariate analysis and analysis after data correction and application of the PSM Propensity Score).

	Univariate OR (CI 95%)	Multivariate * OR (CI 95%)	Adj with PS ** OR (CI 95%)
PREGNANCY, fresh ET (*cIVF vs. ICSI)*	1.064 (0.719–1.574)	1.018 (0.656–1.582)	1.035 (0.674–1.588)
PREGNANCY, fresh+frozen ET *(cIVF vs. ICSI)*	1.064 (0.732–1.545)	1.049 (0.693–1.588)	1.040 (0.695–1.555)
LIVE BIRTH, fresh ET *(cIVF vs. ICSI)*	0.979 (0.609–1.576)	0.881 (0.513–1.514)	0.924 (0.552–1.546)
Single pregnancy (*n* = 116)	0.729 (0.345–1.537)	0.399 (0.143–1.113)	0.692 (0.291–1.644)
Twin pregnancy (*n* = 11)	3.500 (0.145-84.694)	==	==
LIVE BIRTH, fresh+frozen ET *(cIVF vs. ICSI)*	1.096 (0.698–1.723)	0.983 (0.592–1.633)	1.008 (0.619–1.642)
Single pregnancy (*n* = 125)	0.913 (0.430–1.942)	0.457 (0.162–1.289)	0.815 (0.339–1.962)
Twin pregnancy (*n* = 23)	3.500 (0.145–84.694)	==	==

* Adjusted for years of infertility, previous ART treatments, female age, smoke, AFC, AMH, FSH, BMI, endometrial thickness, partner’s age, sperm concentration, progressive motility, normal morphology, embryo morphological score and number of embryos transferred ** Adjusted for Propensity score; CI, confidence intervals; OR, odd ratio.

**Table 4:** Cycle outcome in patients with no previous treatments in the two study groups (univariate analysis).

	cIVF (*n* = 217)	ICSI (*n* = 169)	*p*
Total FSH dose (IU)	2745 ± 1153	2732 ± 1313	0.8456
Mean FSH dose (IU)	262 ± 80	260 ± 88	0.7328
Total LH dose (IU)	1531 ± 797	1578 ± 943	0.9699
Mean LH dose (IU)	191 ± 96	191 ± 63	0.5010
Endometrial thickness (mm)	10.2 ± 2.5	9.9 ± 2.2	0.2969
Retrieved oocytes	5.9 ± 4.0	6.7 ± 4.9	0.3253
Mature (MII) oocytes	4.8 ± 3.1	5.2 ± 3.9	0.9638
MII/retrieved oocytes (%)	85.9 ± 19.1	79.9 ± 19.6	0.0006
Fertilized oocytes	3.5 ± 2.6	3.4 ± 2.9	0.3204
Fertilization rate (%)	59.2 ± 28.9	51.8 ± 28.9	0.0102
No fertilization *(%)*	6.9 (15/217)	11.2 (19/169)	0.1364
Cleaved embryos	3.0 ± 2.5	3.1 ± 2.8	0.8418
Transferred embryos	1.5 ± 0.7	1.4 ± 0.8	0.1070
Frozen embryos	0.4 ± 1.1	0.6 ± 1.3	0.0691
Mean embryo score	8.0 ± 1.8	7.7 ± 1.8	0.1284
PR/ET, *n (%)*	45 (20.7%)	31 (18.3%)	0.5573
Cumulative PR/OPU, *n (%)*	53 (24.4%)	39 (23.1%)	0.7580
LBR/ET, *n (%)*	26 (11.9%)	19 (11.2%)	0.8224
Cumulative LBR/OPU, *n (%)*	32 (14.8%)	22 (13.0%)	0.6271
Abortion rate, *n (%)*	21 (9.7%)	17 (10.1%)	0.9006

**Table 5:** Cycle outcome in patients with no previous treatments in the two study groups (univariate analysis, multivariate analysis and analysis after data correction and application of the PSM Propensity Score).

	Univariate OR (CI 95%)	Multivariate * OR (CI 95%)	Adj with PS ** OR (CI 95%)
PREGNANCY, fresh ET (*cIVF vs. ICSI)*	1.165 (0.700–1.938)	0.906 (0.515–1.594)	1.033 (0.604–1.768)
PREGNANCY, fresh+frozen ET *(cIVF vs. ICSI)*	1.077 (0.671–1.729)	1.049 (0.693–1.588)	0.945 (0.561–1.593)
LIVE BIRTH, fresh ET *(cIVF vs. ICSI)*	0.979 (0.609–1.576)	0.881 (0.513–1.514)	0.924 (0.552–1.546)
Single pregnancy (*n* = 116)	0.729 (0.345–1.537)	0.399 (0.143–1.113)	0.692 (0.291–1.644)
Twin pregnancy (*n* = 11)	3.500 (0.145–84.694)	==	==
LIVE BIRTH, fresh+frozen ET *(cIVF vs. ICSI)*	1.156 (0.644–2.073)	0.895 (0.472–1.696)	0.697 (0.526–1.777)
Single pregnancy (*n* = 125)	1.019 (0.380–2.730)	0.742 (0.140–3.942)	0.800 (0.251–2.555)
Twin pregnancy (*n* = 23)	1.500 (0.055–40.633)	==	==

* Adjusted for years of infertility, previous ART treatments, female age, smoke, AFC, AMH, FSH, BMI, endometrial thickness, partner’s age, sperm concentration, progressive motility, normal morphology, embryo morphological score and number of embryos transferred ** Adjusted for Propensity score.

## Supplementary Materials

The following are available online at https://www.mdpi.com/2077-0383/8/10/1694/s1, Table S1: Basal characteristics of the patients with no previous treatments in the two study groups.

## References

[1] Palermo G., Joris H., Devroey P., Van Steirteghem A.C. (1992). Pregnancies after intracytoplasmic injection of single spermatozoon into an oocyte. Lancet.

[2] Dyer S., Chambers G.M., de Mouzon J., Nygren K.G., Zegers-Hochschild F., Mansour R., Ishihara O., Banker M., Adamson G.D. (2016). International Committee for Monitoring Assisted Reproductive Technologies world report: Assisted Reproductive Technology 2008, 2009 and 2010. Hum. Reprod..

[3] Mansour R., Ishihara O., Adamson G.D., Dyer S., de Mouzon J., Nygren K.G., Sullivan E., Zegers-Hochschild F. (2014). International Committee for Monitoring Assisted Reproductive Technologies world report: Assisted Reproductive Technology 2006. Hum. Reprod..

[4] De Geyter C., Calhaz-Jorge C., Kupka M.S., Wyns C., Mocanu E., Motrenko T., Scaravelli G., Smeenk J., Vidakovic S., Goossens V. (2018). ART in Europe, 2014: Results generated from European registries by ESHRE: The European IVF-monitoring Consortium (EIM) for the European Society of Human Reproduction and Embryology (ESHRE). Hum. Reprod..

[5] Boulet S.L., Mehta A., Kissin D.M., Warner L., Kawwass J.F., Jamieson D.J. (2015). Trends in use of and reproductive outcomes associated with intracytoplasmic sperm injection. JAMA.

[6] Johnson L.N.C., Sasson I.E., Sammel M.D., Dokras A. (2013). Does intracytoplasmic sperm injection improve the fertilization rate and decrease the total fertilization failure rate in couples with well-defined unexplained infertility? A systematic review and meta-analysis. Fertil. Steril..

[7] Tournaye H., Verheyen G., Albano C., Camus M., Van Landuyt L., Devroey P., Van Steirteghem A. (2002). Intracytoplasmic sperm injection versus in vitro fertilization: A randomized controlled trial and a meta-analysis of the literature. Fertil. Steril..

[8] Van Rumste M.M.E., Evers J.L.H., Farquhar C.M. (2004). ICSI versus conventional techniques for oocyte insemination during IVF in patients with non-male factor subfertility: A Cochrane review. Hum. Reprod..

[9] Li Z., Wang A.Y., Bowman M., Hammarberg K., Farquhar C., Johnson L., Safi N., Sullivan E.A. (2018). ICSI does not increase the cumulative live birth rate in non-male factor infertility. Hum. Reprod..

[10] Luna M., Bigelow C., Duke M., Ruman J., Sandler B., Grunfeld L., Copperman A.B. (2011). Should ICSI be recommended routinely in patients with four or fewer oocytes retrieved?. J. Assist. Reprod. Genet..

[11] Sfontouris I.A., Kolibianakis E.M., Lainas G.T., Navaratnarajah R., Tarlatzis B.C., Lainas T.G. (2015). Live birth rates using conventional in vitro fertilization compared to intracytoplasmic sperm injection in Bologna poor responders with a single oocyte retrieved. J. Assist. Reprod. Genet..

[12] Mills M., Rindfuss R.R., McDonald P., te Velde E. (2011). ESHRE Reproduction and Society Task Force Why do people postpone parenthood? Reasons and social policy incentives. Hum. Reprod. Update.

[13] Korkmaz C., Tekin Y.B., Sakinci M., Ercan C.M. (2015). Effects of maternal ageing on ICSI outcomes and embryo development in relation to oocytes morphological characteristics of birefringent structures. Zygote.

[14] Liu D.Y., Garrett C., Baker H.W.G. (2004). Clinical application of sperm-oocyte interaction tests in in vitro fertilization--embryo transfer and intracytoplasmic sperm injection programs. Fertil. Steril..

[15] WHO (2010). WHO Laboratory Manual for the Examination and Processing of Human Semen.

[16] Tannus S., Son W.-Y., Gilman A., Younes G., Shavit T., Dahan M.-H. (2017). The role of intracytoplasmic sperm injection in non-male factor infertility in advanced maternal age. Hum. Reprod..

[17] Holte J., Berglund L., Milton K., Garello C., Gennarelli G., Revelli A., Bergh T. (2007). Construction of an evidence-based integrated morphology cleavage embryo score for implantation potential of embryos scored and transferred on day 2 after oocyte retrieval. Hum. Reprod..

[18] Rosenbaum P.R., Rubin D.B. (1983). The central role of the propensity score in observational studies for causal effects. Biometrika.

[19] Joffe M.M., Rosenbaum P.R. (1999). Invited commentary: Propensity scores. Am. J. Epidemiol..

[20] Abu-Hassan D., Al-Hasani S. (2003). The use of ICSI for all cases of in-vitro conception. Hum. Reprod..

[21] Tucker M., Graham J., Han T., Stillman R., Levy M. (2001). Conventional insemination versus intracytoplasmic sperm injection. Lancet.

[22] Salute M. (2019). Della Relazione del Ministro della Salute al Parlamento Sullo Stato di Attuazione della Legge Contenente Norme in Materia di Procreazione Medicalmente Assistita (L. 19 febbraio 2004, n. 40, articolo 15)-anno. http://www.salute.gov.it/portale/documentazione/p6_2_2_1.jsp?lingua=italiano&id=2866.

[23] Sunderam S., Kissin D.M., Crawford S.B., Folger S.G., Jamieson D.J., Warner L., Barfield W.D. (2017). Assisted Reproductive Technology Surveillance—United States, 2014. MMWR Surveill. Summ..

[24] Check J.H., Chase D.S., Horwath D., Yuan W., Garberi-Levito M.C., Press M. (2012). Oocytes from women of advanced reproductive age do not appear to have an increased risk of zona pellucida hardening. Clin. Exp. Obstet. Gynecol..

[25] Shuai H.-L., Ye Q., Huang Y.-H., Xie B.-G. (2015). Comparison of conventional in vitro fertilisation and intracytoplasmic sperm injection outcomes in patients with moderate oligoasthenozoospermia. Andrologia.

[26] Ebner T., Yaman C., Moser M., Sommergruber M., Jesacher K., Tews G. (2001). A prospective study on oocyte survival rate after ICSI: Influence of injection technique and morphological features. J. Assist. Reprod. Genet..

[27] Rosen M.P., Shen S., Dobson A.T., Fujimoto V.Y., McCulloch C.E., Cedars M.I. (2006). Oocyte degeneration after intracytoplasmic sperm injection: A multivariate analysis to assess its importance as a laboratory or clinical marker. Fertil. Steril..

[28] Farhi J., Cohen K., Mizrachi Y., Weissman A., Raziel A., Orvieto R. (2019). Should ICSI be implemented during IVF to all advanced-age patients with non-male factor subfertility?. Reprod. Biol. Endocrinol..

[29] Sustar K., Rozen G., Agresta F., Polyakov A. (2019). Use of intracytoplasmic sperm injection (ICSI) in normospermic men may result in lower clinical pregnancy and live birth rates. Aust. N. Z. J. Obstet. Gynaecol..

[30] Austin P.C. (2011). An Introduction to Propensity Score Methods for Reducing the Effects of Confounding in Observational Studies. Multivar. Behav. Res..

[31] Martens E.P., Pestman W.R., de Boer A., Belitser S.V., Klungel O.H. (2008). Systematic differences in treatment effect estimates between propensity score methods and logistic regression. Int. J. Epidemiol..

[32] Biondi-Zoccai G., Romagnoli E., Agostoni P., Capodanno D., Castagno D., D’Ascenzo F., Sangiorgi G., Modena M.G. (2011). Are propensity scores really superior to standard multivariable analysis?. Contemp. Clin. Trials.

[33] Weber R.F.A., Dohle G.R., Romijn J.C. (2005). Clinical laboratory evaluation of male subfertility. Adv. Clin. Chem..

[34] Oehninger S., Ombelet W. (2019). Limits of current male fertility testing. Fertil. Steril..

[35] Davies M.J., Moore V.M., Willson K.J., Van Essen P., Priest K., Scott H., Haan E.A., Chan A. (2012). Reproductive technologies and the risk of birth defects. N. Engl. J. Med..

[36] Tararbit K., Lelong N., Thieulin A.-C., Houyel L., Bonnet D., Goffinet F., Khoshnood B. (2013). EPICARD Study Group The risk for four specific congenital heart defects associated with assisted reproductive techniques: A population-based evaluation. Hum. Reprod..

[37] Sandin S., Nygren K.-G., Iliadou A., Hultman C.M., Reichenberg A. (2013). Autism and mental retardation among offspring born after in vitro fertilization. JAMA.

[38] Kissin D.M., Zhang Y., Boulet S.L., Fountain C., Bearman P., Schieve L., Yeargin-Allsopp M., Jamieson D.J. (2015). Association of assisted reproductive technology (ART) treatment and parental infertility diagnosis with autism in ART-conceived children. Hum. Reprod..

[39] Nouri K., Ott J., Stoegbauer L., Pietrowski D., Frantal S., Walch K. (2013). Obstetric and perinatal outcomes in IVF versus ICSI-conceived pregnancies at a tertiary care center—A pilot study. Reprod. Biol. Endocrinol..

[40] Zhu J., Zhu Q., Wang Y., Wang B., Lyu Q., Kuang Y. (2019). Comparative study on risk for birth defects among infants after in vitro fertilization and intracytoplasmic sperm injection. Syst. Biol. Reprod. Med..

[41] Lie R.T., Lyngstadaas A., Ørstavik K.H., Bakketeig L.S., Jacobsen G., Tanbo T. (2005). Birth defects in children conceived by ICSI compared with children conceived by other IVF-methods; a meta-analysis. Int. J. Epidemiol..

[42] Wen J., Jiang J., Ding C., Dai J., Liu Y., Xia Y., Liu J., Hu Z. (2012). Birth defects in children conceived by in vitro fertilization and intracytoplasmic sperm injection: A meta-analysis. Fertil. Steril..

[43] Practice Committees of the American Society for Reproductive Medicine and Society for Assisted Reproductive Technology (2012). Intracytoplasmic sperm injection (ICSI) for non-male factor infertility: A committee opinion. Fertil. Steril..

