# Supplementary material for: ICSI versus Conventional IVF in Women Aged 40 Years or More and Unexplained Infertility: A Retrospective Evaluation of 685 Cycles with Propensity Score Model
Source: J Clin Med. 2019 Oct 16;8(10):1694. doi: 10.3390/jcm8101694 (PMC6833057; doi:10.3390/jcm8101694)
Supplement: Supplementary file 1 [file jcm-08-01694-s001.pdf]

Journal of
Clinical Medicine

MDPI

Table S1. Basal characteristics of the patients with no previous treatments in the two study groups.

	cIVF (*n* = 217)	ICSI (*n* = 169)	*p*
Woman age (years)	41 ± 0.8	41 ± 0.8	0.8825
Years of infertility	2.6 ± 2.0	3.1 ± 2.5	0.0224
BMI (Kg/m^2)	22.7 ± 3.4	23.2 ± 4.1	0.5206
AFC	9.7 ± 5.6	10.5 ± 6.1	0.2100
AMH (ng/mL)	1.6 ± 1.8	1.5 ± 1.4	0.6336
Women who smoke	27 (12.4%)	24 (14.2%)	0.6127
Partner's age (years)	41.9 ± 5	43 ± 5	0.1772
Sperm concentration (Mln/mL)	81 ± 33.3	75.2 ± 35.8	0.1029
Progressive motility (A+B; %)	40.6 ± 4.9	39.9 ± 4.3	0.0849
Normal morphology (%)	5.0 ± 0.8	5 ± 0.8	0.6329

BMI, body mass index; AFC, antral follicular count; AMH, anti-Mullerian hormone; cIVF, conventional in vitro fertilization; ICSI, Intracytoplasmic sperm injection.
